# A New Medical Journal

**Published:** 1853-01

**Authors:** 


### THE WESTERN JOURNAL.

THIRD SERIES.—VOL. XI.—NO. I.

LOUISVILLE, JANUARY 1, 1853.

### A NEW MEDICAL JOURNAL.

What, *another Medical Journal!* will be the exclamation, we fancy, of many a reader, as his eye falls upon the above caption. Yes, gentle reader; it is proposed to add another to the long catalogue of American medical serials. Before us is the prospectus of the "*Virginia Medical and Surgical Journal*," of which Drs. George A. Otis and H. L. Thomas, of Richmond, are to be the editors. The first number of the journal is to appear in April, and thereafter it is to be issued in monthly numbers of 80 large octavo pages, at $5. a year. Each number will be composed in part of a portion of some standard work in one of the departments of medicine. "The objects proposed in its publication are the diffusion of medical knowledge and intelligence, the advancement of science, and the support of the dignity and interests of the profession." We like the tone and style of the prospectus, and, from the place and terms of its publication, are free to say that we believe the Virginia journal will be true to these objects. We shall look with interest for its appearance, and in advance assure its editors of our best wishes fo their success.

Since the above was written, we have received from Nashville the first number of the "*Southern Journal of the Medical and Physical Sciences,*" edited by Drs. King, Jones, Curry, and Wood. It is to appear bi-monthly, at $2. a year, and will form an annual volume of 432 pages. The first number is well printed, on good paper, and makes a very creditable appearance. It contains articles from each of its editors, together with the usual proportion of selected matter. A peculiarity of this new candidate for public favor, is the number of its editors and the distribution amongst them of the several departments of the profession; Drs. King and Jonestaking Practical Medicine, Dr. Curry, Chemistry and Pharmacy, and Dr. Wood, Dental Surgery. We incline to believe that this arrangement is judicious, and that it gives to the enterprise amid the great competition of the times increased chances for success.

But is it possible that all the journals in existence and contemplated, can live? Are they and will they be supported? We are no prophets and shall not attempt to foretell what will be the fate of many of our respected contemporaries; but we are sure we are fully warranted in saying, that at the present time their support is most inadequate. Hardly one comes to us in which there are not complaints that subscribers do not pay well. The cover of our own journal bears too frequent testimony to the tardiness with which the demands of our publishers are met. As a body, we take it that the medical editors of the United States labor for a smaller pecuniary remuneration than any other class of men in the community. The hope of such reward cannot be a motive to these enterprises.

The rapidity with which medical, literary, and political journals have been multiplied in our country during the present century, is one of the marvels of our wonderful age. Less than a century and a half ago, there was not a newspaper published in America. The Pilgrims had been nearly a hundred years in New England before the first, which was the Boston News Letter, was set on foot. Fifteen years afterwards one was started in Philadelphia, and six years later the first in New York was issued. South Carolina was without one until 1730, and the first were printed in Connecticut and New Hampshire less than a century ago. Now, "their name is legion"—they are thick as the leaves in Vallambrosa. If we do not, at this time, sustain a more prolific newspaper presst han all the other nations of the earth besides, it is certain that we have far more newspapers than any single nation in the world; and the proportion of our medical journals is fully as great.

It was near the close of the last century when our first periodical devoted to medicine was establised, and at that time there were few such journals in existence anywhere. The *Medical Repository* was begun at New York in 1798, and for fifteen years had no ally or rival in our country. From first to last, in fact, it may be said to have occupied the field alone, for while it went on to the completion of its twenty-third volume, its few contemporaries, after struggling on for a year or two, were discontinued for want of patronage. The *Medical Recorder* succeeded to the popularity of the Medical Repository, and thirty years ago, if not the only one in existence, was the only one that was widely circulated, or had any considerable duration. Another was at length established by its side in Philadelphia; and this still goes on under a new name, the largest, the most elaborate, and the best supported of all our professional serials. The *Boston Medical and Surgical Journal* in age ranks next to the *American Journal of the Medical Sciences*, and its accomplished and amiable editor, we believe, has been longer on the tripod than any of his brethren, and is therefore the patriarch of the fraternity.

Looking over our exchange list, we find that the number of our medical journals has now swelled to twenty eight, not to speak of those devoted to Pharmacy, Insanity, Dentistry, and the Sciences allied to medicine, or of that mongrel brood which defiles while professing to reform and advance our noble profession. Of these New York furnishes five, Pennsylvania four, Tennessee four, Ohio two, Louisiana two, Kentucky two, and Massachusetts, New Hampshire, New Jersey, Virginia, South Carolina, Georgia, Missouri, Iowa, and Illinois, each, one. All France and Great Britain together scarcely do more than this.

We have remarked, that most of these publications afford but too indubitable proof of insufficient pecuniary support. Most of them barely pay expenses, if, indeed, they are not a tax upon their conductors; and in this connection another question naturally suggests itself—*is their literary support any better?* We believe that no one, after a careful inspection of the weekly and monthly issues of our medical press, will answer this question in the affirmative. The pages of our journals testify to a sad deficiency of practised, competent contributors. Any one who will be at the trouble to estimate the proportion of "original" matter in them, one month with another, will find that it is small, and, what is worse, that the articles forming this department have too probably obtained admission, in many cases, for no better reason than the straitness of the editor's supplies. A journal once launched forth must be continued to the end of the year, whether it has subscribers or not, and its pages must be filled with or without the aid of contributors. Publishing day comes round and the editor is not prepared for it; he racks his brains for matter, but discovers, at length, that his powers of production have their limits. His Hippocrene has run dry. In despair he seizes upon whatever may chance to lie within his reach, re-touches, or remodels it, vamps it up, and sends it forth among his "original" essays and cases.

We have often asked ourselves the question, where is this tending, and in what is it to end? There are certainly more journals than are demanded, and the number is not likely soon to grow less, but rather to increase. Few have been discontinued within the last ten years, and during that period one at least has been added annually to the list. But this very excess is attended with some advantages, one of which is, and it is a most important one, that it fosters and develops in our profession a taste and talent for writing. Hundreds of articles are prepared for the periodicals of the neighborhood, which would never be sent to a journal at a distance, and writers are thus found and brought out who otherwise would have spent their days in obscurity. In this way, too, the whole profession of the country is laid under contribution and its experience placed upon record. Cases of rare interest, facts having important practical bearings, are brought to light by this searching process which, in the absence of the competition, would probably remain unrecorded. And what though the history of them be given in uncouth phrases and an unpolished style? The function of the editor is to correct and improve these, and so that the matter be good, he can afford to be indulgent as to the rest.

We once heard of a correspondent of a western journal who, on the appearance of his first article in print, wrote a kind letter to the editor, in which he related the following anecdote: He had once a friend, he said, who concluded to buy a little horse at auction because he was going cheap—an ill-formed, starved, sorry looking little creature. He sent him to the country to be recruited. After a few weeks his pony was brought back to him, but so metamorphosed that he did not recognize him. He could hardly be persuaded that the plump, sleek animal before him was the shabby thing that he had a little while before dispatched to the country. We may add, that this correspondent was not discouraged by the liberties which the editor took with his manuscript, but has continued to write, and now when his papers return to him he has no difficulty in recognizing them. We have no doubt but the experience of our brethren of the quill would furnish many similar instances. But all correspondents are not so reasonable, nor so improvable. What for example, could be made of a man who wrote thus?

"Dear Sir I wish you to Stop sending the Medical Journal to me for I wiil leave for oregon this Spring So close up for it is the no a countest thing I ever read it is at least 50 years behind the times you had better stop grinding or turn Eclectic then you will be a benefit to mankind

M. JEFFERIES M. D."

We think this rare literary specimen is worth preserving, and doubt much whether any of our brethren can furnish from their correspondence any thing to match it. *Dr. Jefferies*, it will be remarked, does not deign to make a stop in his letter. It is to be hoped that he did not stop till he got to Oregon.

In what we have written, it has been far from our purpose to repress the ardor of any of our friends about embarking in the business of editorship. We would rather applaud their spirit and cheer them on to the work. Tendering to them and to all our brethren—the editors of the old journals and of the new—of those in *posse* as well as of those in *Esse*—the compliments of the season, we wish them abundant success in their labors.

